# Predictive Accuracy of the 1‐h HACOR Score for Identifying Non‐Invasive Ventilation Failure in COPD Patients With Acute Hypercapnic Respiratory Failure: A Prospective Observational Study

**DOI:** 10.1002/hsr2.73312

**Published:** 2026-09-29

**Authors:** Md. Joynal Abedin, Md. Zakir Hossain Sarker, Md. Ferdous Wahid, Md. Mahbub Islam, Mohammed Mirazur Rahman, S. M. Masuduzzaman

**Affiliations:** ^1^ Department of Respiratory Medicine Chittagong Medical College Chittagong Bangladesh; ^2^ Department of Respiratory Medicine National Institute of Diseases of the Chest & Hospital Dhaka Bangladesh; ^3^ Department of Respiratory Medicine Labaid Cardiac Hospital Dhaka Bangladesh; ^4^ Department of Respiratory Medicine Kushtia Medical College Hospital Kushtia Bangladesh

**Keywords:** acute exacerbation of chronic obstructive pulmonary disease, acute hypercapnic respiratory failure, chronic obstructive pulmonary disease, HACOR score, non‐invasive ventilation

## Abstract

**Background and Aims:**

Chronic obstructive pulmonary disease (COPD) is frequently complicated by acute hypercapnic respiratory failure (AHRF) requiring non‐invasive ventilation (NIV). Early identification of NIV failure is important, as delayed recognition may worsen outcomes. This study evaluated the predictive utility of the 1‐h HACOR score for early identification of NIV failure in COPD patients.

**Methods:**

A prospective observational study was conducted at the National Institute of Diseases of the Chest and Hospital, Dhaka, from May 2022 to September 2023. Ninety‐two patients of acute exacerbations of COPD with AHRF were purposively enrolled. Clinical parameters and HACOR scores were recorded at baseline and 1 h after NIV initiation. NIV failure was defined as death or need for intubation, classified as early (< 48 h) or late (≥ 48 h).

**Results:**

About 13.1% patients experienced NIV failure. The HACOR score decreased significantly at 1 h of NIV initiation in the success group (8.16 ± 4.52 to 3.38 ± 2.93; *p* < 0.001) but remained persistently high in the failure group. A HACOR cut‐off value of ≥ 6 at 1 h predicted NIV failure with 91.67% sensitivity, 83.75% specificity, while ≥ 8 reliably identifies patients at high risk of early NIV failure with 100% sensitivity and 89.29% specificity.

**Conclusions:**

The HACOR score was a simple and reliable bedside tool for early prediction of NIV failure in patients with acute exacerbation of COPD complicated by AHRF. Its routine use may facilitate timely reassessment and escalation of respiratory support, although larger multicenter studies are needed for further validation.

## 1 Introduction

Chronic obstructive pulmonary disease (COPD) ranks as the fourth leading cause of death globally, with an estimated 3.5 million deaths in 2021, accounting for nearly 5% of all deaths worldwide [1]. COPD patients, on average, suffer from acute exacerbation, defined as sudden worsening of symptoms beyond day‐to‐day variability that requires additional treatment—are frequently driven by risk factors, comorbidities, poor adherence, and inadequate therapy. [2, 3, 4]. Despite the high risks associated with COPD exacerbations—where hypercapnic respiratory failure (HRF) occurs in 20% of cases and mortality can reach 33%—the use of non‐invasive ventilation (NIV) offers a vital means of stabilizing patients and avoiding invasive intubation [5, 6]. NIV diminishes the effort required for respiration, enhances arterial oxygenation and alveolar ventilation, obviates the necessity for invasive mechanical ventilation, lowers the incidence of ventilator‐associated pneumonia, shortens the duration of intensive care unit admission, and reduces mortality rates [7, 8]. Because NIV failure is linked to higher mortality and longer hospital stays than NIV success, the delayed identification of failing patients can lead to late intubation and invasive ventilation, further increasing morbidity and mortality [9, 10]. To facilitate timely clinical decisions between invasive ventilation, conservative care, or palliation, it is imperative to identify predictive markers of NIV failure early, thereby ensuring prompt intubation when necessary [9].

An elevated APACHE II score (> 20.5) upon presentation is a strong predictor of NIV failure; nevertheless, because of its complexity, this indicator is hardly employed outside critical care environments [10, 11]. Alongside standardized scoring, researchers have discerned various demographic and metabolic indicators of failure, such as younger age, diminished arterial bicarbonate levels, reduced PaCO_2_, elevated arterial lactate, baseline anemia, suboptimal WHO Performance Status, and a Nutritional Risk Screening (NRS 2002) score exceeding 3 [10, 12, 13].

Clinical presentation is a key determinant of prognosis, as NIV failure is commonly associated with tachycardia, acidosis, depressed consciousness, impaired oxygenation, and increased respiratory rate [9, 14, 15, 16, 17, 18, 19, 20]. Although these individual variables are beneficial for monitoring, it is crucial to acknowledge that the predictive capability for failure is limited when depending on any single indicator alone; a multifactorial evaluation is necessary for clinical precision [21]. Researchers employed stepwise multivariable regression analysis to identify critical parameters assessed 1 h post‐NIV commencement that effectively predict failure. This resulted in the creation of the HACOR score, which allocates points to five distinct variables: heart rate, acidosis (pH), consciousness (Glasgow Coma Scale [GCS]), oxygenation (PaO_2_/FiO_2_), and respiratory rate.

The HACOR score integrates five bedside variables—heart rate, acidosis (pH), consciousness (GCS), oxygenation (PaO_2_/FiO_2_), and respiratory rate—to estimate the risk of NIV failure, with higher scores indicating greater risk [21]. The original HACOR score was developed in patients with acute hypoxemic respiratory failure, while a subsequently derived COPD‐specific version retained the same five components but used different weighting of individual variables to predict NIV failure in COPD patients with HRF [21, 22]. Its components are readily available and facilitate early bedside assessment. Previous studies have demonstrated good predictive performance of the HACOR score for NIV failure in patients with acute hypoxemic respiratory failure, including assessment within 1–2 h of NIV initiation [21, 23]. Evidence in COPD patients has similarly supported its usefulness for early prediction of NIV failure, particularly for failure occurring within the first 48 h [22]. Alongside appropriate ventilator settings, close monitoring, and multidisciplinary care, early risk assessment using tools such as the HACOR score may support timely clinical decision‐making during NIV therapy [22].

Although the HACOR score has demonstrated promising predictive performance in patients with acute hypoxemic respiratory failure, evidence specific to acute exacerbations of COPD with acute hypercapnic respiratory failure (AHRF) remains limited, particularly in low‐ and middle‐income countries. Differences in patient characteristics, disease severity, and healthcare settings may influence its predictive performance. A standardized reassessment at 1 h after NIV initiation may provide an early opportunity for risk stratification. Therefore, this study aimed to evaluate the predictive performance of the 1‐h HACOR score for NIV failure among patients with COPD with AHRF. The findings may provide preliminary evidence regarding its potential role as an adjunct to early clinical assessment, while further multicenter validation would be required before routine clinical implementation.

## 2 Methods

A prospective observational study was conducted in the Respiratory Intensive Care Unit and Respiratory Care Unit of the National Institute of Diseases of the Chest and Hospital (NIDCH), Dhaka, from May 2022 to September 2023. Formal ethical approval for the study was obtained from the ethical review committee of NIDCH [Ref: NIDCH/ERC/2023/267].

Patients presenting to the study units with acute exacerbation of COPD who required NIV were assessed for eligibility. The sample size was calculated a priori using a diagnostic accuracy approach based on the anticipated sensitivity (93.9%) and specificity (88.6%) of the HACOR score reported by Duan et al. [22]. Considering a 95% confidence level, 10% absolute precision, and an expected NIV failure prevalence of 24.0% reported by Confalonieri et al. [24], the calculated sample sizes were 92 based on sensitivity and 52 based on specificity. The larger value was selected, resulting in a required sample size of 92 participants.

A total of 108 patients were assessed for eligibility. Patients aged > 40 years with acute exacerbation of COPD, arterial blood pH < 7.35, and PaCO_2_ > 50 mmHg who required NIV were considered eligible for inclusion. Of the 108 patients assessed, 16 were excluded: 3 required emergency intubations, 2 had NIV intolerance, 4 had other obstructive airway diseases, 3 had coexisting interstitial lung disease, 1 had chest deformity, 1 had a neurological disease contributing to ventilatory failure, and 2 declined participation or were excluded for other reasons. Purposive sampling was used to recruit patients who met the predefined clinical and arterial blood gas criteria for AHRF requiring NIV during the study period. Accordingly, 92 eligible patients were enrolled and included in the final analysis. No sedative or anxiolytic medication was administered specifically to facilitate NIV tolerance; patient comfort and compliance were optimized through appropriate mask fitting, reassurance, close monitoring, and adjustment of ventilator settings.

A pretested data collection sheet was used for data collection. Patient demographic characteristics, comorbidities, and risk behavior, HACOR score (before NIV initiation and 1 h after NIV), and NIV outcome (success/failure) were noted. The HACOR score represented the aggregate of points assigned to the five variables. The score ranges from 0 to 27, with elevated values signifying an increased probability of NIV failure [22]. NIV failure was defined as the occurrence of endotracheal intubation or death during NIV therapy [24]. Early NIV failure was defined as failure occurring within 48 h of NIV initiation, whereas failure occurring beyond 48 h of NIV support was classified as late NIV failure [25].

At admission, before initiating the HACOR, parameters were taken. The patients were managed under the close supervision of attending doctors or consultants, along with the nursing staff. NIV was administered in accordance with the hospital's standard protocol. The preferred interface was the oro‐nasal mask, and the mask size was appropriately adjusted to fit the individual's facial structure. The ventilation mode employed was bi‐level positive airway pressure. To optimize dyspnea control and ensure patient comfort, the initial inspiratory positive airway pressure was set at 12 cm H_2_O and then gradually increased as needed. Similarly, the expiratory positive airway pressure started at 4 cm H_2_O and was adjusted upward based on clinical response. The fraction of inspired oxygen (FiO_2_) was titrated to maintain a partial pressure of arterial oxygen (PaO_2_) above 60 mmHg and to keep peripheral oxygen saturation (SpO_2_) above 90% on bedside pulse oximetry.

One hour after initiation of NIV, clinical and laboratory parameters, including arterial blood pH, PaO_2_, PaCO_2_, PaO_2_/FiO_2_ ratio, GCS, respiratory rate, and heart rate, were reassessed. The 1‐h response was used to evaluate the early effectiveness of NIV rather than to initiate weaning. Patients showing clinical and physiological improvement continued NIV with periodic reassessment and adjustment of ventilator settings as required. Weaning from NIV was considered subsequently after sustained clinical improvement and the ability to maintain adequate spontaneous respiration. Patients who showed clinical deterioration or fulfilled predefined criteria for endotracheal intubation were escalated to invasive mechanical ventilation. Nonetheless, the patients were monitored until discharge to evaluate the ultimate outcome. The timing and date of the commencement of invasive mechanical ventilation have been documented for subjects who underwent intubation.

Endotracheal intubation was indicated in the presence of persistent respiratory distress with a respiratory rate exceeding 35 breaths per minute, inability to maintain a PaO_2_/FiO_2_ ratio above 100 mmHg, failure to correct respiratory acidosis, neurological deterioration requiring airway protection (such as coma or seizure activity), excessive airway secretions, hemodynamic instability refractory to fluid resuscitation and vasoactive therapy, or the occurrence of respiratory or cardiac arrest.

A participant flow diagram has been added as Figure 1, showing the number of patients assessed for eligibility, excluded with reasons, enrolled, and included in the final analysis, together with their final NIV outcomes.

**Figure 1:**
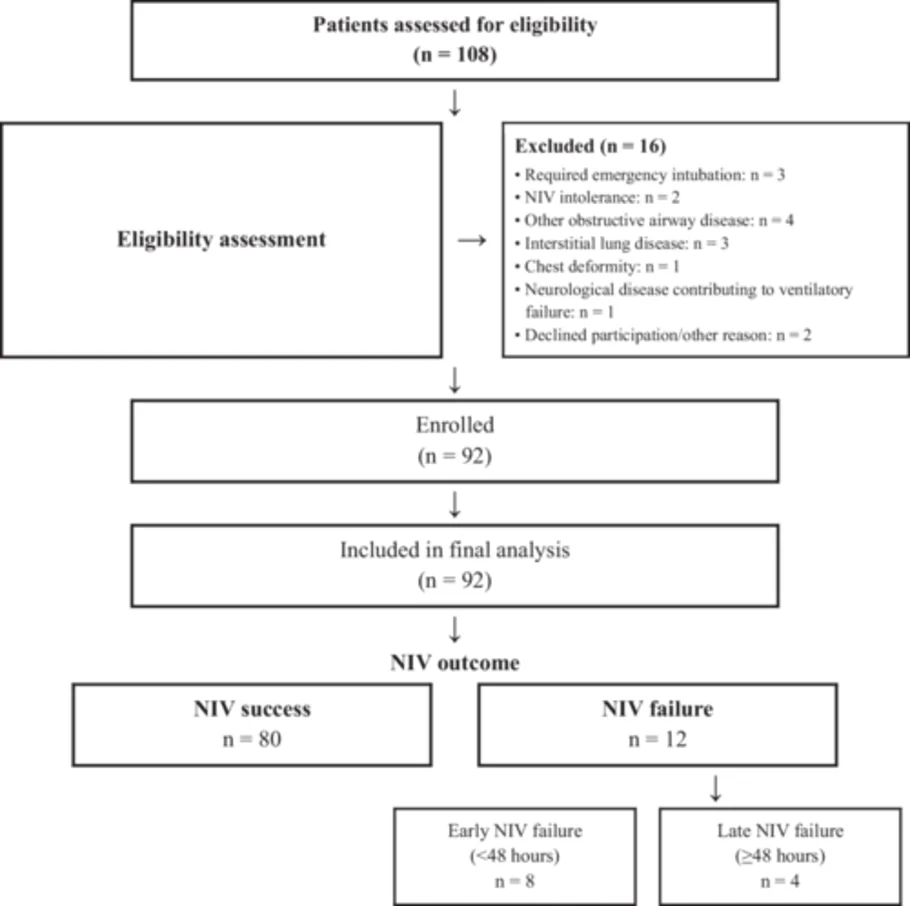
Flow of participants through the study. NIV, non‐invasive ventilation.

## 3 Statistical Analysis

Following data collection, data were entered, checked for completeness, accuracy, and consistency, and analyzed using IBM SPSS Statistics for Windows, version 23.0 (IBM Corp., Armonk, NY, USA). Data normality was assessed using the Shapiro–Wilk test. Continuous variables were summarized as mean ± standard deviation or median (range), as appropriate, while categorical variables were presented as frequencies and percentages. Categorical variables were compared using the chi‐square test or Fisher's exact test, as appropriate. HACOR scores between the NIV success and failure groups were compared using the independent‐samples *t*‐test for normally distributed data or the Mann–Whitney *U*‐test for non‐normally distributed data. Changes in HACOR scores from baseline to 1 h after NIV initiation were assessed using the Wilcoxon signed‐rank test. The predictive performance of the 1‐h HACOR score for NIV failure was evaluated using receiver operating characteristic (ROC) curve analysis. The area under the ROC curve (AUC) with 95% confidence interval (CI) was calculated to assess discriminatory ability. The optimal cut‐off value was determined using the Youden index, and the corresponding sensitivity, specificity, positive predictive value (PPV), negative predictive value (NPV), and overall diagnostic accuracy were calculated. Separate ROC analyses were performed for early NIV failure (< 48 h) and late NIV failure (≥ 48 h). All statistical tests were two‐sided, and a *p* value < 0.05 was considered statistically significant.

## 4 Results

Among the 92 patients enrolled in the study, NIV was successfully implemented in 86.9% (*n* = 80) of cases. Conversely, NIV failure was observed in 13.1% (*n* = 12) of the study population. The majority experienced early failure (*n* = 8; 8.7%), while a smaller subset experienced late failure (*n* = 4; 4.4%) (Figure 2).

**Figure 2:**
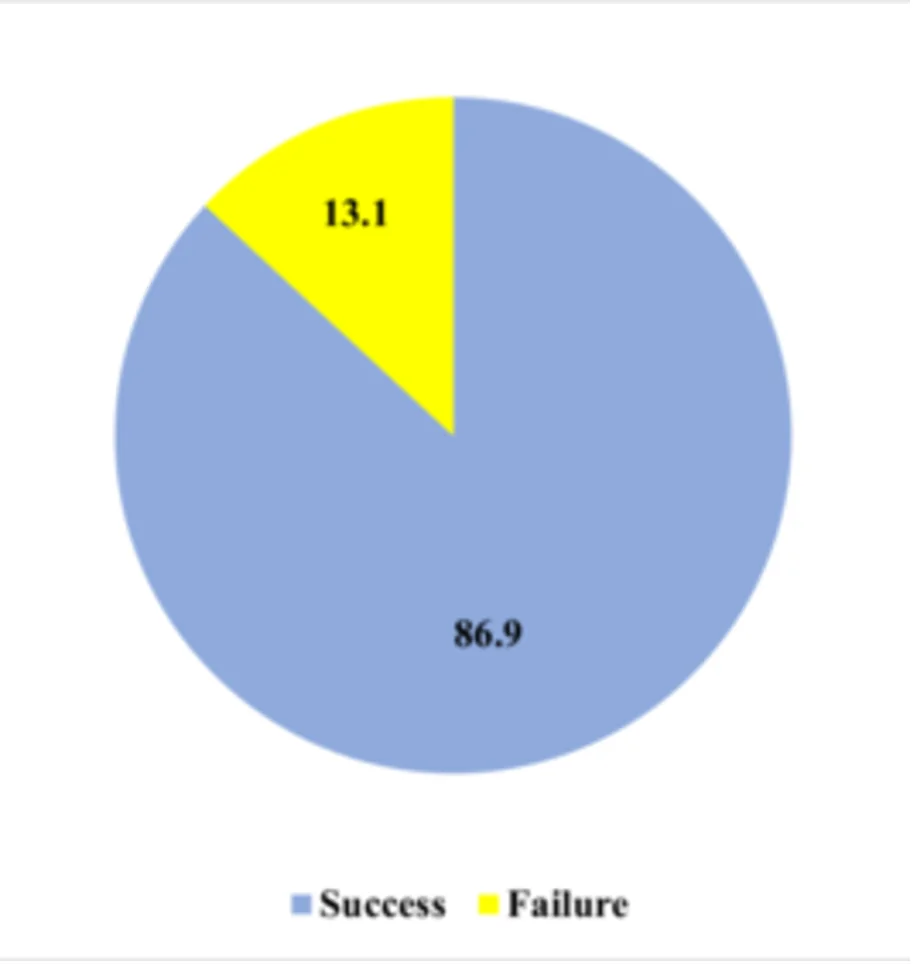
Distribution of non‐invasive ventilation outcomes of COPD patients (*N* = 92).

The baseline demographic characteristics were well‐balanced between the two outcome groups, indicating that these factors did not significantly impact the clinical success of NIV in this study (Table 1). Among the clinical comorbidities evaluated, chronic kidney disease was significantly associated with NIV failure, occurring in 33.3% of the failure group compared to 8.8% of the success group (*p* = 0.034). Other conditions, such as ischemic heart disease (39.1%), hypertension (21.7%), and diabetes mellitus (14.1%), were common but did not significantly impact outcomes. While underweight status was more prevalent in the failure group (50.0% vs. 21.2%), the difference did not reach statistical significance (*p* = 0.08). Similarly, smoking status, with a high prevalence of ex‐smokers (79.3%), showed no significant correlation with NIV success or failure (*p* = 0.276) (Table 2). Analysis of clinical and arterial blood gas parameters showed significant differences between the NIV success and failure groups, both at baseline and following initiation. Before NIV initiation, the failure group presented with significantly higher PaCO_2_ (98.03 vs. 82.15 mmHg; *p* = 0.029), more severe acidosis (pH: 7.23 vs. 7.29; *p* = 0.002), and lower GCS scores (12.25 vs. 13.41; *p* = 0.01) compared to the success group. Baseline heart rate, respiratory rate, and PaO_2_/FiO_2_ ratio did not differ significantly between the groups (*p* > 0.05) (Table 3). Following the first hour of NIV, the disparity between groups became more pronounced. Significant differences were observed in pH (7.33 vs. 7.25; *p* < 0.001), GCS (14.58 vs. 12.00; *p* < 0.001), PaO_2_/FiO_2_ ratio (192.15 vs. 130.42; *p* = 0.001), and respiratory rate (23.56 vs. 26.17 breaths per min; *p* = 0.009). Persistent hypercapnia remained a feature of the failure group (PaCO_2_: 87.13 vs. 70.64 mmHg; *p* = 0.028) (Table 3). The NIV success group showed a highly significant clinical improvement within the first hour, with mean HACOR scores dropping from 8.16 ± 4.52 to 3.38 ± 2.93 (*p* < 0.001). In contrast, the NIV failure group exhibited persistently high scores that showed no significant reduction after 1 h of therapy (12.33 ± 3.17 vs. 11.92 ± 4.87; *p* = 0.579) (Table 4). ROC analysis of HACOR score at 1 h of NIV initiation to predict NIV failure, with an AUC value of 0.931 (95% CI: 0.856–1.00), which was statistically significant (*p* < 0.001) (Figure 3). A HACOR score cut‐off of ≥ 6 showed the best predictive performance, with the highest Youden index (0.754), a sensitivity of 91.67% (95% CI: 61.52%–99.79%), and a specificity of 83.75% (95% CI: 73.82%–91.05%). The overall diagnostic accuracy was 84.78%, with an NPV of 98.53% and a PPV of 45.83% (Figure 4). ROC analysis demonstrated excellent discrimination of the 1‐h HACOR score for early NIV failure (< 48 h), with an AUC of 0.967 (95% CI: 0.931–1.000; *p* < 0.001). Discrimination for late NIV failure (≥ 48 h) was also good, with an AUC of 0.842 (95% CI: 0.664–1.000; *p* = 0.021) (Figure 5). At a cut‐off of ≥ 8, the HACOR score predicted early NIV failure with 100.0% sensitivity, 89.3% specificity, and an overall accuracy of 90.2%. For late NIV failure, a cut‐off of ≥ 6 yielded a sensitivity of 75.0%, specificity of 83.8%, and overall accuracy of 83.3%, indicating lower but acceptable predictive performance compared with early NIV failure (Table 5).

**Table 1:** Distribution of the study participants according to demographic characteristics (*N* = 92).

Demographic characteristics	NIV outcome		Total	*p* value
	Success (*n*_1_ = 80)	Failure (*n*_2_ = 12)		
Age (years), mean ± SD	60.36 ± 8.65	63.25 ± 8.54	60.5 ± 8.61	0.516^b^
Gender				
Male	77 (96.3)	12 (100.0)	89 (96.7)	> 0.99^c^
Female	3 (3.8)	0 (0.0)	3 (3.3)	
Occupation				
Day laborer	7 (8.8)	0 (0.0)	7 (7.6)	0.486^a^
Cultivator	22 (27.5)	3 (25.0)	25 (27.2)	
Service holder	9 (11.3)	0 (0.0)	9 (9.8)	
Businessmen	13 (16.3)	2 (16.7)	15 (16.3)	
Housewife	3 (3.8)	0 (0.0)	3 (3.3)	
Retired	15 (18.8)	5 (41.7)	20 (21.7)	
Others	11 (13.8)	2 (16.7)	13 (14.1)	

*Note:* Data were presented as frequency, percentage, mean ± SD, and median (range).

Abbreviations: NIV, non‐invasive ventilation; SD, standard deviation.

^a^ Chi‐square test.

^b^ Student's *t*‐test.

^c^ Fisher's exact test.

**Table 2:** Distribution of the participants according to comorbidity and risk behaviors in different NIV outcomes (*N* = 92).

Comorbidity and risk behaviors	NIV outcome		Total	*p* value
	Success (*n*_1_ = 80)	failure (*n*_2_ = 12)		
Diabetes mellitus	11 (13.8)	2 (16.7)	13 (14.1)	0.676^a^
Hypertension	16 (20.0)	4 (33.3)	20 (21.7)	0.285^a^
Ischemic heart disease	30 (37.5)	6 (50.0)	36 (39.1)	0.528^a^
Chronic kidney disease	7 (8.8)	4 (33.3)	11 (12.0)	0.034^a^
Chronic liver disease	2 (2.5)	0 (0.0)	2 (2.2)	> 0.99^a^
Smoking				
Smoker	14 (17.5)	0 (0.0)	14 (15.2)	0.276^b^
Ex‐smoker	62 (77.5)	11 (91.7)	73 (79.3)	
Non smoker	4 (5.0)	1 (8.3)	5 (5.4)	
Body mass index				
Underweight (< 18.5 kg/m^2^)	17 (21.2)	6 (50.0)	23 (25.0)	0.08^b^
Normal weight (18.5–24.9 kg/m^2^)	56 (70.0)	6 (50.0)	62 (67.4)	
Overweight (25.0–29.9 kg/m^2^)	7 (8.8)	0 (0.0)	7 (7.6)	

*Note:* Data were presented as frequency, percentage.

Abbreviation: NIV, non‐invasive ventilation.

^a^ Fisher's exact test.

^b^ Chi‐square test.

**Table 3:** Vital signs and ventilator parameters before NIV initiation and at 1 h of NIV initiation.

Time frame				NIV outcome		Total	*p* value
	Parameters			Success (*n*_1_ = 80)	Failure (*n*_2_ = 12)		
Before NIV initiation	PaCO_2_		Mean ± SD	82.15 ± 20.07	98.03 ± 22.53	84.25 ± 20.98	0.029^a^
			Median (range)	82 (51–141.70)	92 (65–142.0)	84 (51–142)	
	HACOR parameters	Heart rate (per minute)	Mean ± SD	103.68 ± 16.54	103.33 ± 22.11	103.64 ± 17.24	0.754^a^
			Median (range)	107 (64–138)	95 (76–136)	106 (64–138)	
		pH	Mean ± SD	7.29 ± 0.05	7.23 ± 0.063	7.28 ± 0.05	0.002^a^
			Median (range)	7.3 (7.1–7.34)	7.24 (7.08–7.30)	7.2 (7.08–7.34)	
		GCS	Mean ± SD	13.41 ± 1.56	12.25 ± 1.06	13.25 ± 1.55	0.01^a^
			Median (range)	14 (10–15)	12 (11–15)	13 (10–15)	
		PaO_2_/FiO_2_	Mean ± SD	153.51 ± 83.14	125.08 ± 80.41	149.76 ± 82.91	0.09^a^
			Median (range)	128 (63–660)	95.5 (58–360)	122 (58–660)	
		Respiratory rate (per minute)	Mean ± SD	26.08 ± 5.10	27.75 ± 4.90	26.31 ± 5.1	0.273^a^
			Median (range)	24 (18–34)	28 (20–34)	26 (18–34)	
1 h of NIV initiation	PaCO_2_		Mean ± SD	70.64 ± 18.35	87.13 ± 26.45	72.79 ± 20.20	0.028^a^
			Median (range)	71.1 (39.90–119)	94.6 (40–122)	72.1 (39.90–122)	
	HACOR parameters	Heart rate (per minute)	Mean ± SD	95.03 ± 14.80	101.67 ± 18.68	95.90 ± 15.41	0.161^b^
			Median (range)	96 (64–133)	95 (82–132)	96 (64–133)	
		pH	Mean ± SD	7.33 ± 0.05	7.25 ± 0.05	7.32 ± 0.05	< 0.001^a^
			Median (range)	7.33 (7.19–7.43)	7.25 (7.20–7.32)	7.32 (7.19–7.43)	
		GCS	Mean ± SD	14.58 ± 0.89	12.00 ± 1.48	14.24 ± 1.31	< 0.001^a^
			Median (range)	15 (11–15)	12 (10–15)	15 (10–15)	
		PaO_2_/FiO_2_	Mean ± SD	192.15 ± 60.36	130.42 ± 46.11	184.01 ± 62.12	0.001^a^
			Median (range)	201 (65–335)	113.5 (81–232)	191 (65–335)	
		Respiratory rate (per minute)	Mean ± SD	23.56 ± 4.35	26.17 ± 1.99	23.91 ± 4.20	0.009^a^
			Median (range)	22 (18–34)	26 (22–28)	22 (18–34)	

*Note:* Data were presented as mean ± SD, median (range)

Abbreviations: FiO_2_, fraction of inspired oxygen; GCS, Glasgow Coma Scale; NIV, non‐invasive ventilation; PaCO_2_, partial pressure of arterial carbon dioxide; PaO_2_, partial pressure of oxygen; SD, standard deviation.

^a^ Mann–Whitney *U*‐test.

^b^ Student's *t*‐test.

**Table 4:** HACOR score before NIV initiation and at 1 h of NIV initiation.

Non‐invasive ventilation (NIV)		HACOR score		*p* value
		Before NIV	At 1 h of NIV	
Success (*n*_1_ = 80)	Mean ± SD	8.16 ± 4.52	3.38 ± 2.93	< 0.001^a^
	Median (range)	8 (1–19)	3 (0–14)	
Failure (*n*_2_ = 12)	Mean ± SD	12.33 ± 3.17	11.92 ± 4.87	0.579^a^
	Median (range)	13 (5–16)	12 (3–19)	

*Note:* Data were presented as mean ± SD, median (range).

Abbreviations: NIV, non‐invasive ventilation; SD, standard deviation.

^a^ Wilcoxon signed rank test.

**Figure 3:**
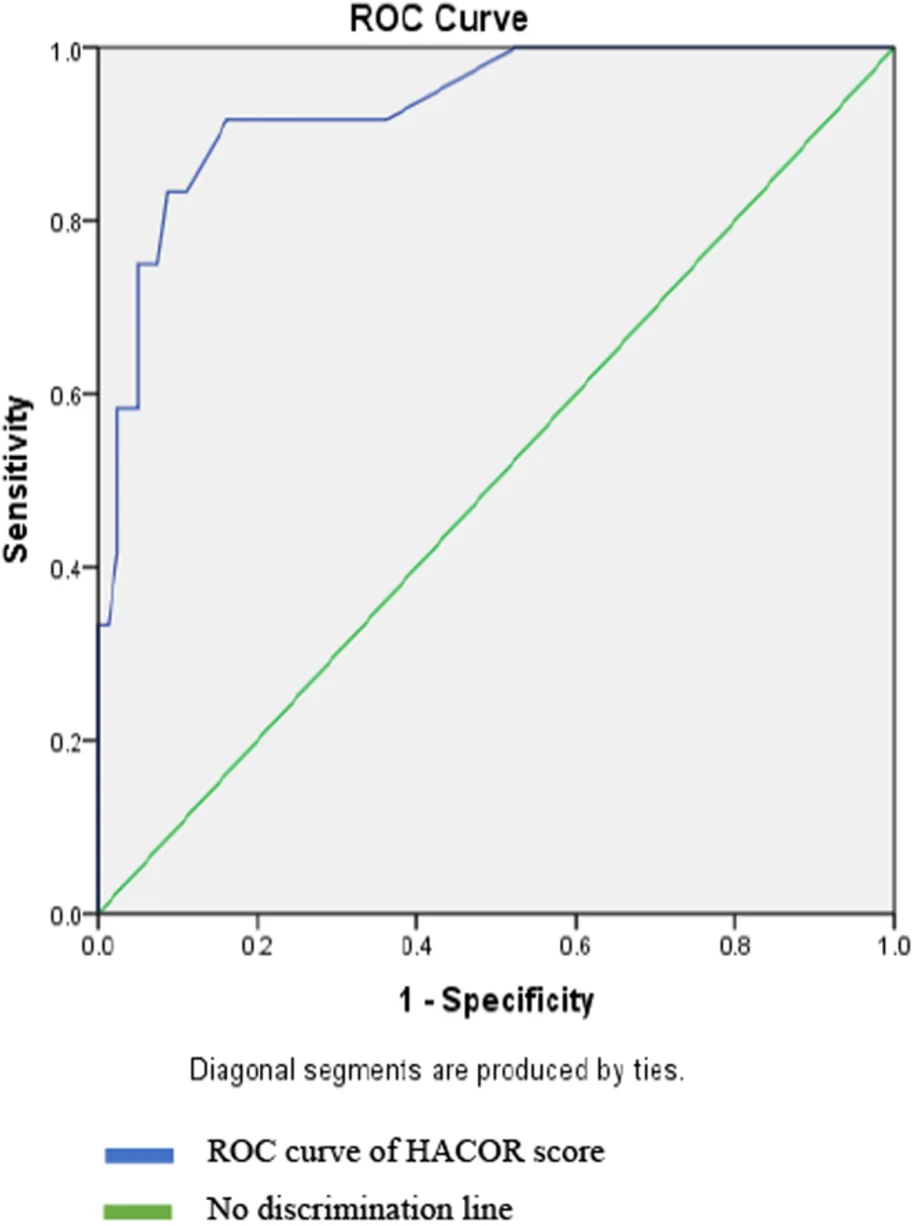
ROC curve of HACOR score to early predict NIV failure.

**Figure 4:**
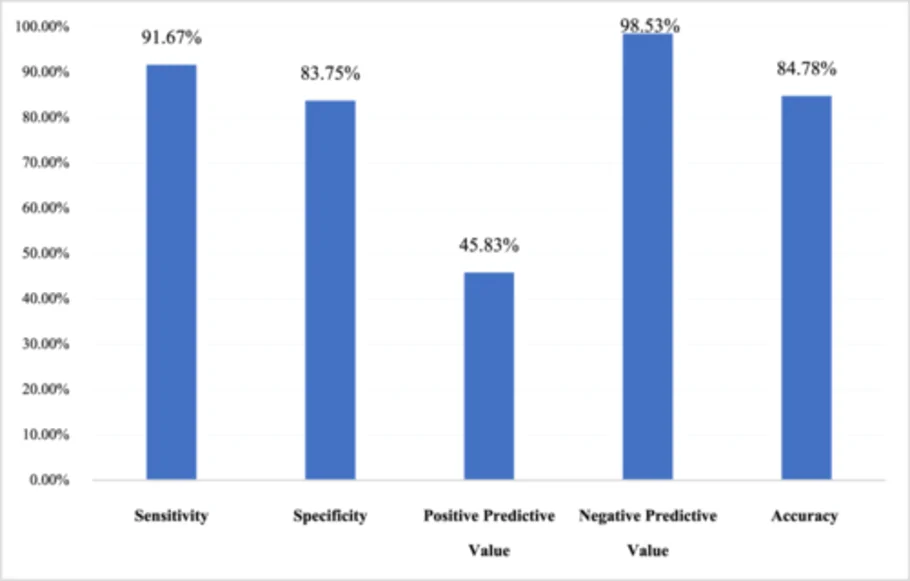
Sensitivity, specificity, positive predictive value, negative predictive value, and accuracy gained by the derived cut‐off value for early predicting NIV failure.

**Figure 5:**
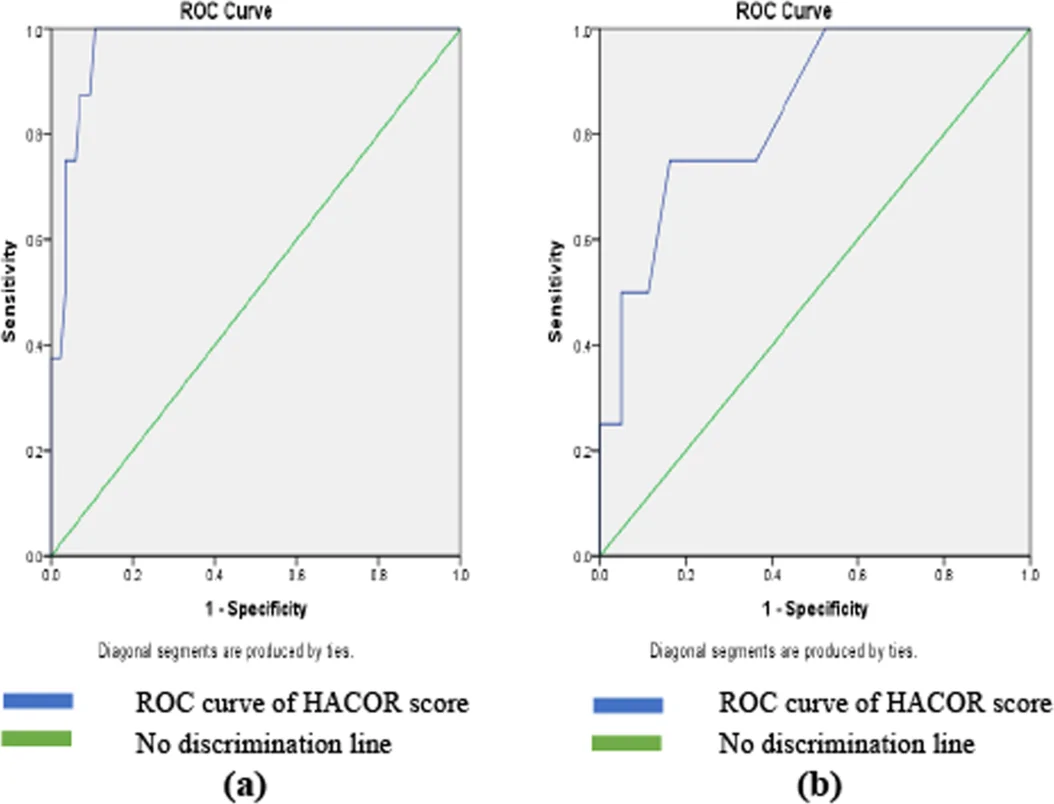
ROC curve of HACOR score to predict (a) early NIV failure and (b) late NIV failure.

**Table 5:** Sensitivity, specificity, positive predictive value, negative predictive value, and accuracy gained by the derived cut‐off value for predicting early and late NIV failure.

	Cut‐off	Sensitivity	Specificity	PPV	NPV	Accuracy
Early NIV failure	≥ 8	100.00%	89.29%	47.06%	100.00%	90.22%
Late NIV failure	≥ 6	75.00%	83.75%	18.75%	98.53%	83.33%

Abbreviations: NIV, non‐invasive ventilation; NPV, negative predictive value; PPV, positive predictive value.

## 5 Discussion

As a leading cause of mortality worldwide, COPD commonly progresses to acute respiratory failure necessitating ventilatory support. Although NIV is often the first‐line therapy, its failure necessitates endotracheal intubation—a transition associated with poor clinical prognosis. Reducing morbidity and mortality hinges on the early prediction of treatment failure. This study evaluated the predictive utility of the HACOR score and demonstrated good discriminatory performance for the early identification of NIV failure, which may support more timely clinical decision‐making.

In the present study, the NIV failure rate was lower than that reported in the derivation and internal‐validation cohorts of Duan et al. [22], but higher than that observed in their external‐validation cohort. Differences in patient populations, underlying disease severity, NIV management strategies, and criteria used to define NIV failure may account for this variation across studies. The absence of a significant association between age and NIV outcome was consistent with the findings of Steriade et al. [27], Duan et al. [21], and Duan et al. [26], suggesting that age may not be a major determinant of NIV success or failure. Similarly, the comparable distribution of gender and occupation between the NIV success and failure groups suggests that these baseline characteristics were unlikely to have substantially influenced NIV outcomes in the present cohort.

Duan et al. [22] found that heart rate, acidosis (assessed by pH), consciousness (assessed using the GCS), oxygenation, and respiratory rate were associated with NIV failure, which were the variables that constitute the HACOR score. In this study, we found that at baseline, except for heart rate and respiratory rate, other variables such as acidosis, consciousness, and oxygenation were significantly associated with NIV failure. But after 1 h of NIV, except heart rate and, others variable like, respiratory rate, acidosis, consciousness, and oxygenation were significantly associated with NIV failure. In this study, a significant difference in HACOR between NIV success and failure. Duan et al. [22] also reported that HACOR score significantly varies between success and failure group. A major challenge in acute COPD management is the early identification of NIV failure. While the predictive chart by Confalonieri et al. [24] is accurate, its reliance on the APACHE II score makes it cumbersome for real‐time assessment. The HACOR score overcomes these barriers by utilizing easily obtainable bedside variables. This efficiency is vital, as it provides high predictive power within 1–2 h of initiation—a timeframe where laboratory data is often incomplete. Consequently, HACOR serves as a superior, streamlined scoring system for guiding immediate clinical interventions.

A HACOR score of ≥ 6 demonstrated good discriminatory performance for NIV failure in the present study, with findings broadly consistent with those reported by Duan et al. [22], who identified a similar threshold and observed a comparable NIV failure rate among patients with higher scores. This consistency supports the potential utility of the HACOR score for early risk stratification during NIV. The predictive value of the score may be attributable to its incorporation of multiple physiological and clinical parameters that reflect the patient's early response to NIV. From a clinical perspective, a high score may alert clinicians to patients requiring closer monitoring and early reassessment for potential escalation of respiratory support.

The utility of the HACOR score extends beyond patients with COPD. Carrillo et al. [23] validated the score in hypoxemic patients and demonstrated that assessment 1 h after NIV initiation could identify patients at risk of NIV failure, particularly in conditions such as pneumonia and ARDS. The consistent performance observed in our study, despite the use of a slightly different threshold (≥ 6 vs. > 5), further supports the applicability of the HACOR score across different patient populations and clinical settings. In particular, its high sensitivity and negative predictive value indicate potential value in early risk stratification, as patients with lower scores are less likely to experience NIV failure. This may help clinicians identify patients who are more likely to continue NIV successfully while prioritizing closer monitoring and early reassessment for those with higher scores.

The 1‐h HACOR score also showed potential utility for identifying patients at risk of early NIV failure. A higher score was strongly associated with imminent NIV failure, with a score of ≥ 8 identifying all patients who subsequently experienced early failure. Although not all patients with a score ≥ 8 developed early NIV failure, the high sensitivity and specificity observed in this cohort support the potential role of this threshold in early risk stratification. In clinical practice, a low HACOR score may provide reassurance regarding the likelihood of early NIV success, whereas a higher score may prompt closer monitoring and earlier reassessment of the need for escalation of respiratory support.

These findings are broadly comparable to those reported by Duan et al. [22], who identified a HACOR score > 5 as an optimal threshold for predicting early NIV failure. The slightly higher cut‐off observed in the present study may reflect differences in patient characteristics, disease severity, timing of NIV initiation, and healthcare delivery across study settings. In our setting, limited availability of NIV facilities, variation in staff expertise, and delayed presentation may contribute to greater physiological derangement at the time of NIV initiation. Such contextual differences may influence the early HACOR score and consequently the optimal threshold for identifying patients at risk of NIV failure.

The HACOR score also demonstrated useful discriminatory performance for late NIV failure, indicating that the score assessed shortly after NIV initiation may provide information beyond immediate treatment response. Taken together, the findings suggest that a score ≥ 6 at 1 h may be useful for early risk stratification of overall NIV failure, whereas the higher threshold of ≥ 8 may provide additional value in identifying patients at greater risk of early NIV failure among COPD patients with acute respiratory failure. This distinction may help clinicians tailor the intensity of monitoring and reassess the need for escalation of respiratory support according to the patient's early response to NIV.

In summary, the 1‐h HACOR score demonstrated good discriminatory performance for risk stratification of NIV failure in COPD patients with AHRF. A threshold of ≥ 6 showed utility for identifying patients at risk of overall NIV failure, while a higher threshold of ≥ 8 was particularly useful for identifying those at greater risk of early NIV failure. The simplicity and bedside applicability of the score may support early clinical risk assessment and closer monitoring, potentially facilitate timely reassessment and escalation of respiratory support when clinically indicated. However, these findings should be interpreted in the context of the limited number of NIV failure events and the inability to perform robust multivariable adjustment.

## 6 Limitations

This study has several limitations. First, its single‐center design and purposive sampling may have introduced selection bias and limited the generalizability of the findings to other clinical settings. Second, the relatively small sample size and, particularly, the limited number of NIV failure events reduced statistical precision and may have resulted in unstable or optimistic estimates of diagnostic performance and derived cut‐off values, especially in the early‐ and late‐failure subgroup analyses. The small number of outcome events also precluded reliable multivariable logistic regression because of the risk of overfitting and unstable estimates. Furthermore, several clinically relevant variables, including pH, GCS, oxygenation, and respiratory rate, are components of the HACOR score itself; therefore, simultaneous adjustment for these variables and the total HACOR score would have been inappropriate because of substantial overlap and potential multicollinearity. Consequently, residual confounding cannot be excluded, and the observed associations should not be interpreted as evidence of an independent predictive effect. Third, HACOR scores were assessed only at baseline and 1 h after NIV initiation, preventing evaluation of serial changes in the score over time. In addition, long‐term outcomes and other potentially relevant factors, such as comorbidity severity, were not assessed.

## 7 Conclusions

NIV was successful in most patients with acute exacerbation of COPD with AHRF, whereas NIV failure, particularly late failure, was associated with higher mortality. The HACOR score assessed 1 h after NIV initiation showed good discriminatory ability for predicting NIV failure. A threshold of ≥ 6 was useful for identifying patients at risk of overall NIV failure, while a higher threshold of ≥ 8 identified those at greater risk of early failure. Changes in the HACOR score during the first hour may provide additional information on early treatment response and help identify patients requiring closer monitoring and timely reassessment. Given its simplicity and bedside applicability, the 1‐h HACOR score may be particularly useful in resource‐constrained settings. However, it should be considered an adjunct to clinical assessment rather than a stand‐alone criterion for escalation to invasive ventilation. Given the limited number of NIV failure events and the inability to perform robust multivariable adjustment, these findings and proposed thresholds should be interpreted cautiously and validated in larger, multicenter prospective studies.

## Author Contributions

Md. Joynal Abedin conceived and designed the study, data acquisition, performed analysis and interpretation of data, drafted the manuscript, and critically revised the manuscript for important intellectual content. Md. Zakir Hossain Sarker contributed to study design, supervised data acquisition, analysis of data, critical revision of the manuscript for important intellectual content, and provided overall supervision. Md. Ferdous Wahid contributed to the study design, participated in data acquisition, analysis and interpretation of data, and drafting of the manuscript. Md. Mahbub Islam contributed to the study design, analysis and interpretation of data, and critically revised the manuscript. Mohammed Mirazur Rahman contributed to study design, analysis and interpretation of data, and critically revised the manuscript. S. M. Masuduzzaman contributed to study design, analysis and interpretation of data, and critically revised the manuscript. All authors have read and approved the final version of the manuscript. Md. Joynal Abedin, the corresponding author, had full access to all of the data in this study and takes complete responsibility for the integrity of the data and the accuracy of the data analysis.

## Funding

The authors have nothing to report.

## Ethics Statement

This study was conducted in accordance with the ethical principles outlined in the Declaration of Helsinki. Ethical approval was obtained from the ethical review committee of the National Institute of Diseases of the Chest and Hospital (NIDCH), Dhaka, Bangladesh [Ref: NIDCH/ERC/2023/267; Date: 14.5.2022]. Written informed consent was obtained from the legally authorized attendants of all participants prior to enrollment. Confidentiality and anonymity of participants were strictly maintained throughout the study.

## Conflicts of Interest

The authors declare no conflicts of interest.

## Transparency Statement

Md. Joynal Abedin, the manuscript guarantor, affirms that this manuscript is an honest, accurate, and transparent account of the study being reported; that no important aspects of the study have been omitted; and that any discrepancies from the study as planned.

## Data Availability

The data supporting the findings of this study are available from the corresponding author upon reasonable request. Due to ethical considerations and institutional policies regarding patient confidentiality, the raw data are not publicly available.
